# Implantable cardioverter defibrillator therapy in paediatric patients for primary vs. secondary prevention

**DOI:** 10.1093/europace/euae245

**Published:** 2024-09-30

**Authors:** Jani Thuraiaiyah, Berit Thornvig Philbert, Annette Schophuus Jensen, Lucas Yixi Xing, Troels Hoejsgaard Joergensen, Chee Woon Lim, Frederikke Noerregaard Jakobsen, Pernille Steen Bække, Michael Rahbek Schmidt, Lars Idorn, Morten Holdgaard Smerup, Jens Brock Johansen, Sam Riahi, Jens Cosedis Nielsen, Ole De Backer, Lars Sondergaard, Christian Jons

**Affiliations:** Department of Cardiology, Copenhagen University Hospital, Rigshospitalet, Inge Lehmanns Vej 7, 2100 Copenhagen, Denmark; Department of Cardiology, Copenhagen University Hospital, Rigshospitalet, Inge Lehmanns Vej 7, 2100 Copenhagen, Denmark; Department of Cardiology, Copenhagen University Hospital, Rigshospitalet, Inge Lehmanns Vej 7, 2100 Copenhagen, Denmark; Department of Cardiology, Copenhagen University Hospital, Rigshospitalet, Inge Lehmanns Vej 7, 2100 Copenhagen, Denmark; Department of Cardiology, Copenhagen University Hospital, Rigshospitalet, Inge Lehmanns Vej 7, 2100 Copenhagen, Denmark; Department of Cardiology, Copenhagen University Hospital, Rigshospitalet, Inge Lehmanns Vej 7, 2100 Copenhagen, Denmark; Department of Cardiology, Odense University Hospital, Odense, Denmark; Department of Cardiology, Copenhagen University Hospital, Rigshospitalet, Inge Lehmanns Vej 7, 2100 Copenhagen, Denmark; Department of Cardiology, Copenhagen University Hospital, Rigshospitalet, Inge Lehmanns Vej 7, 2100 Copenhagen, Denmark; Department of Paediatrics, Copenhagen University Hospital, Rigshospitalet, Copenhagen, Denmark; Department of Cardiothoracic Surgery, Copenhagen University Hospital, Rigshospitalet, Copenhagen, Denmark; Department of Cardiology, Odense University Hospital, Odense, Denmark; Department of Cardiology, Aalborg University Hospital, Aalborg, Denmark; Department of Clinical Medicine, Aalborg University, Aalborg, Denmark; Department of Cardiology, Aarhus University Hospital, Aarhus, Denmark; Department of Clinical Medicine, Aarhus University, Aarhus, Denmark; Department of Cardiology, Copenhagen University Hospital, Rigshospitalet, Inge Lehmanns Vej 7, 2100 Copenhagen, Denmark; Abbott Structural Heart, Santa Clara, CA, USA; Department of Cardiology, Copenhagen University Hospital, Rigshospitalet, Inge Lehmanns Vej 7, 2100 Copenhagen, Denmark

**Keywords:** Implantable cardioverter defibrillator, Appropriate therapy, Paediatric cardiology, Congenital heart disease, Sudden cardiac death

## Abstract

**Aims:**

The decisions about placing an ICD in a child are more difficult than in an adult due to longer expected lifespan and the complication risk. Young patients gain the most years from ICDs, despite higher risk of device-related complications. The secondary prevention ICD indication is clear, and device is implanted regardless of potential complications. For primary prevention, risk of sudden cardiac death and complications need to be evaluated. We aimed to compare outcomes for primary and secondary prevention ICDs.

**Methods and results:**

Retrospective nationwide cohort study including paediatric patients identified from the Danish ICD registry with ICD implanted at an age ≤ 15 from 1982–21. Demographics, complications (composite of device-related infections or lead-failure requiring re-operation, mortality because of arrhythmia, or unknown cause), and mortality were retrieved from medical charts. Endpoint was appropriate therapy (shock or anti-tachycardia pacing for ventricular tachycardia or fibrillation). Of 72 receiving an ICD, the majority had channelopathies (*n* = 34) or structural heart diseases (*n* = 28). ICDs were implanted in 23 patients for primary prevention and 49 for secondary prevention, at median ages of 13.8 and 11.6 years (*P*-value 0.01), respectively. Median follow-up was 9.0 (interquartile ranges: 4.7–13.5) years. The 10-year cumulative incidence of first appropriate therapy was 70%, with complication and inappropriate therapy rates at 41% and 15%, respectively. No difference was observed between prevention groups for all outcomes. Six patients died during follow-up.

**Conclusion:**

In children, two-thirds are secondary prevention ICDs. Children have higher appropriate therapy and complication rates than adults, while the inappropriate therapy rate was low.

## Graphical Abstract

**Graphical Abstract:**
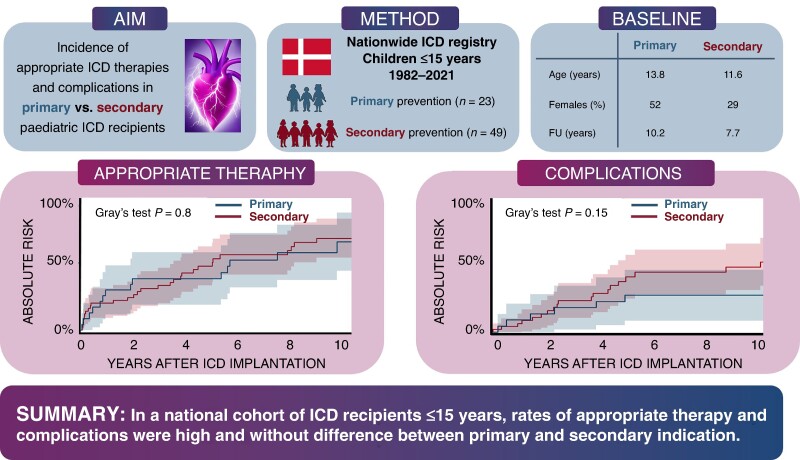

**What’s new?**

- There is a high and similar rate of appropriate ICD therapies in patients with ICDs for primary and secondary prevention.
- ICD-related complications are significant and comparable between primary and secondary prevention recipients.

## Introduction

Our understanding of the optimal utilization of implantable cardioverter defibrillator (ICD) in paediatric patients is limited, although the incidence of ICD implantations in these patients is increasing.^1–3^ For a child surviving cardiac arrest or with a documented malignant ventricular arrhythmia, ICD implantation is indicated as secondary prevention.^4^ However, in paediatric patients who are suspected to be at high risk of arrhythmic death, but without documented malignant arrhythmia, the timing of ICD implantation for primary prevention is still uncertain.^5,6^

Among Danish adults, the number of primary and secondary recipients is nearly equal.^7^ Primary prevention is indicated in adults with ischaemic or non-ischaemic cardiomyopathy accompanied by severely reduced ejection fraction and symptoms of heart failure, or in patients with either congenital heart diseases, hypertrophic cardiomyopathies, dilated cardiomyopathies, or arrhythmogenic cardiomyopathies with a high-risk profile.^8^ However, risk assessment is more challenging in children due to heterogenicity with different risk score models for different aetiologies.^4,9^ Most published papers on paediatric ICDs include adults up to the age of 21 years,^1,2,5,10–15^ and there is a paucity of literature supporting the decision to implant an ICD for primary prevention in high-risk paediatric patients below the age of 16 years. In addition, ICD implantation for primary prevention in the paediatric age group requires careful consideration including individual risk stratification for sudden cardiac death (SCD), which is thoroughly discussed with the family before a final decision is made. The following parameters are included in the decision to implant an ICD for primary prevention: clinical presentation including syncope or heart failure, channelopathies, family history, or structural heart disease. However, data about the effectiveness of this patient selection process are lacking.^4^

This study aimed to describe and compare the indications for ICD implantation, rates of ICD therapies, and the clinical courses of Danish paediatric patients ≤ 15 years old who received ICD for primary or secondary prevention.

## Methods

### Data source

The present study used data from the Danish Pacemaker and ICD Registry (DPIR) and medical charts. DPIR is a national database established in 1982 including all implanting centres in Denmark.^16^ DPIR contains detailed clinical and technical information on all cardiac implantable electronic device procedures. The registry is controlled by the Danish Society of Cardiology with a steering committee that represents all centres in Denmark. Patients were followed from the first ICD implantation until either emigration or death. Each Danish citizen is assigned a unique civil registration number enabling comparison across Danish registries. This unique number can be crossmatched with the Danish National Patient Register, which contains data on all hospital admissions, outpatient visits, procedures, and operations. All data were validated from medical charts.

### Patient population

All patients aged ≤15 years at the time of index ICD implantation up to 2021 were identified from DPIR. Subsequently, patient characteristics, device characteristics, device therapies, and ICD-related complications were retrieved from medical charts.

### Approvals

The study was approved by the Danish Patient Safety Authority (no.: 31-1521-398) and the institutional review board. The study adhered to the ethical guidelines of the Declaration of Helsinki. Informed consent from patients was not required due to the retrospective design of the study.

### Definitions and data collection

ICD implanted without documented spontaneous ventricular arrhythmias were defined as *primary prevention*.

*Secondary prevention* was defined as ICD implantation after either documented sustained ventricular tachycardia (VT), ventricular fibrillation (VF), or clinically aborted cardiac arrest.

If the patient had symptoms suspicious of ventricular arrhythmia and after a thorough examination including genetic testing, exercise testing, imaging, and electrophysiologic examination revealed an inducible ventricular arrhythmia, the implantation was classified as secondary.^17,18^

### Outcome

Data were collected for the following outcomes: appropriate and inappropriate therapies, complications, and mortality.

Appropriate therapy was defined as shock or anti-tachycardia pacing for ventricular tachycardia or fibrillation. Complication was defined as the composite of device-related infections and lead-related outcomes requiring re-operation including lead fracture, increased threshold, perforations, displacements, and mortality because of arrhythmia or of unknown cause. Complications were further divided into procedural (≤30 days) or post-procedural (>30 days after implantation).

The primary outcome consisted of time to first appropriate ICD therapy. The incidence of appropriate therapy was assessed as recurrent events allowing multiple events per subject during follow-up. Further, complications, inappropriate shock therapy, and mortality were assessed as separate outcomes.

### Follow-up

For each patient, follow-up time was defined as the time from index ICD implantation to the following: outcome of interest, heart transplantation with ICD extraction, death, emigration, or end of study defined as 26 January 2021.

### Statistics

For baseline analysis, continuous variables are presented as medians with interquartile ranges (IQR) and compared using the Kruskal–Wallis test. For age and weight, ranges are presented. Categorial variables are presented as frequencies with percentages, and χ^2^ test was applied to compare the distributions. All outcomes were analysed as time-to-first-event. The survival rate was determined by the Kaplan–Meier estimator and compared with the log-rank test. For all other outcomes than mortality, cumulative incidences were reported using Aalen–Johansen estimator with death as a competing risk and compared with Gray’s test. Relative risks of outcomes were estimated using cause-specific Cox-regression models. The proportional hazards assumption was evaluated with scaled Schoenfeld residuals, and no violation was found.

For appropriate therapy, in addition to time-to-first-event analysis, the relative risk of total incidence was assessed using the Andersen–Gill model accounting for recurrent events in the same subject.^19^ In these analyses, age was assessed as a categorical variable using the median value of the cohort as the cut-off and as a continuous variable. The groupwise difference in covariance was tested using the Kruskal–Wallis test. A two-tailed *P*-value of <0.05 was considered statistically significant. All data were analysed using R (version 4.1.2).

## Results

### Demographics

From 1982 to 2021, a total of 72 patients aged ≤15 years (range 0–15 years) received their index ICD implantation, corresponding to 39 ICDs pr. million live births. For primary prevention, an ICD was implanted in 23 patients, whereas for secondary prevention, 49 patients were implanted with an ICD. Clinical indications are presented in *Table 1*, and clinical characteristics are presented in *Table 2*. The median age at index implantation was higher for primary prevention than for secondary prevention group (13.8 years (IQR 12.7–14.2) vs. 11.6 years (IQR 8.4–14.0), *P* < 0.05). The weight ranged from 5.8 to 70 kg. There were no differences in the distribution of the underlying cardiac diagnosis for the two prevention groups. The majority of the cohort had channelopathies (*n* = 34, 47%), followed by structural heart diseases (*n* = 28, 39%). All ICDs for primary prevention and 39 (80%) for secondary prevention were implanted transvenously. For the epicardial implantations, the ICD coil was most often implanted in the transverse sinus or posteriorly on left thorax below the scapula. Epicardial dual-coils (*n* = 4) were tunnelled posterior to the superior vena cava and into the transverse sinus. Placement was secured with suture to the pericardium, taking care not to injure or impinge upon the right phrenic nerve. The heart was immersed in saline to allow for electrical conduction, and the direct current function was tested after induction of VF with diathermy. Alternatively, if transverse sinus placement was deemed unsuitable because of pericardial adherences or because no sternotomy was made, coils were tunnelled subcutaneously to a position on the back near the scapula (*n* = 4). The pace-sense electrodes were placed as apical as possible on the right ventricle and/or left ventricle epicardium as appropriate depending upon the accessibility and whether there was visible myocardium. Furthermore, they were placed in a manner so as not to damage or impinge upon the left coronary artery and its branches. The surgical note for the last child with epicardial implantation was not retrievable to describe details about the placement of coils. The child died within one year of ICD implantation. Median weight at index implantation for the patients with secondary indication and epicardial implantation was 15.8 kg. Single-chamber systems were implanted in 62 children, while dual-chamber systems were implanted in 9 children besides the one child with a subcutaneous implantable cardioverter-defibrillator (S-ICD). This child was of adequate weight (>45 kg) and had a body composition with substantial subcutaneous fat. For this particular child, S-ICD seemed reasonable, so the child and family were informed about the choice between transvenous and S-ICD and favoured S-ICD. A dual-chamber ICD was implanted in children with either a pacing indication or known long QT syndrome. In cases of arrhythmia, beta-blockers, flecainide, mexiletine, and amiodarone were administered. If a long duration of therapy was expected, amiodarone was avoided. In case of heart failure, beta-blockers and angiotensin-converting enzyme inhibitors were administered. In recipients with either catecholaminergic polymorphic ventricular tachycardia (CPVT) or congenital long QT syndrome, *n* = 26 (76%) were treated with beta-blockers (see Supplementary material online, *S1*).

**Table 1:** Indication for ICD implantation

	Number of patients
Primary prevention recipients	27
Pre-syncope or syncope with gene-verified channelopathy	14
Pre-syncope or syncope with structural heart disease	4
Symptoms highly suspicious of arrhythmia and inducible monomorphic VT^a^	4
Structural heart disease with symptomatic heart failure, with either syncope or positive family history	3
Syncope and positive family history	1
Syncope despite ablation for nsVT	1
Secondary prevention recipients	45
VF or cardiac arrest	38
Monomorphic or polymorphic VT	7
Total	72

nsVT, non-sustained ventricular tachycardia; VF, ventricular fibrillation; VT, ventricular tachycardia.

^a^These four patients were classified as primary prevention recipients in a sensitivity analysis and classified as secondary prevention recipients in the main analysis.

**Table 2:** Baseline characteristics of all patients receiving ICD implantation from 1982 to 2021

	All	Primary prevention	Secondary prevention	*P*-value
Number of patients	72	23	49	<0.001
Female	26 (36%)	12 (52%)	14 (29%)	0.05
Height, cm^a^	157 (136–165)	161 (155–166)	151 (135–164)	0.07
Weight, kg^a^	42.0 (31.0–52.0)	48 (40.5–57.1)	39.4 (29.5–47.6)	0.02
Age at implantation, year	13.0 (10.0–14.1)	13.8 (12.7–14.2)	11.6 (8.4–14.0)	0.01
Underlying diagnosis				0.38
Channelopathy	34 (47%)	13 (57%)	21 (43%)	
Congenital long QT	22	11	11	
CPVT	12	2	10	
Structural heart disease	25 (35%)	8 (35%)	17 (35%)	
Congenital heart disease				
Aortic stenosis	1	0	1	
AVSD	1	1	0	
Pulmonary stenosis	1	0	1	
Single ventricle	1	0	1	
TGA	1	0	1	
TOF	2	0	2	
Truncus arteriosus communis	2	1	1	
VSD	1	0	1	
Cardiomyopathies				
ARCV	5	3	2	
Dilated cardiomyopathy	4	2	2	
Hypertrophic cardiomyopathy	6	1	5	
Idiopathic VF	5 (7%)	0 (0%)	5 (10%)	
Ischaemic heart disease	1 (1%)	0 (0%)	1 (2%)	
Other	7 (10%)	2 (9%)	5 (10%)	
Anti-arrhythmic drug				0.80
Beta-blocker	43 (59.7%)	16 (59.3%)	27 (60.0%)	
Digoxin	1 (1.4%)	1 (3.7%)	0 (0%)	
Isoptin	1 (1.4%)	1 (3.7%)	0 (0%)	
Follow-up (years)	9.0 (4.7–13.5)	10.2 (6.6–12.4)	7.7 (3.6–15.3)	0.20
Implantation technique				0.07
Transvenous	62 (86%)	23 (100%)	39 (80%)	
Epicardial	9 (13%)	0 (0%)	9 (18%)	
Subcutaneous	1 (1%)	0 (0%)	1 (2%)	

Continuous variables are presented as medians with interquartile ranges, whereas categorical variables are presented as counts with percentages.

ARVC, arrhythmogenic right ventricular cardiomyopathy; AVSD, atrioventricular septal defect; CPVT, catecholaminergic polymorphic ventricular tachycardia; TGA, transposition of the great arteries; TOF, tetralogy of Fallot; VF, ventricular fibrillation; VSD, ventricular septal defect.

^a^Seven patients had missing height, while five had missing weight.

**Figure 1:**
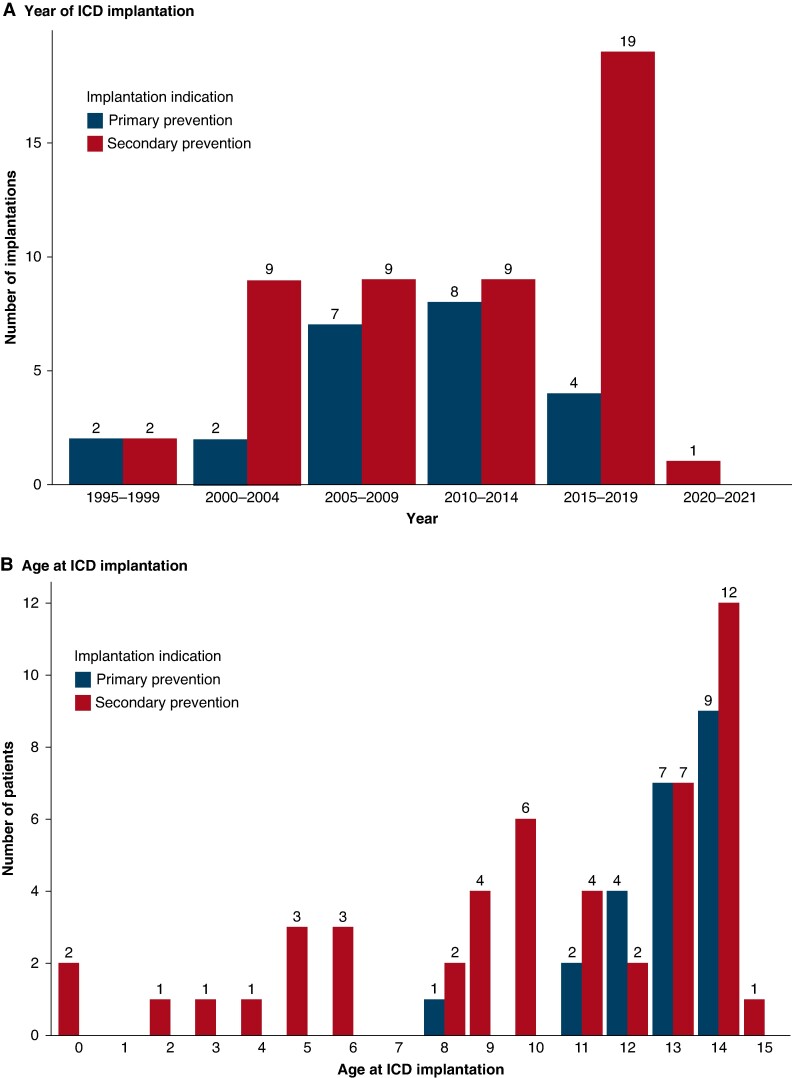
Characteristics of ICD implantation. (*A*) Number of patients undergoing ICD implantation stratified by prevention group. (*B*) Distribution of ICD implantations by age at the time of implantation. ICD, implantable cardioverter defibrillator.

The number of index ICD implantations increased since 1995 as depicted in *Figure 1A*. Especially from 2015 to 2019, the secondary prevention ICDs increased. The cohort underwent 153 interventions over a median follow-up period of 9 years. These included 72 (47%) first implantations, 39 (25%) elective replacement indicators with the change of generators, 31 (21%) complications, 5 (3%) revisions, 2 (1%) system upgrades, and 4 (3%) extractions due to no further indication e.g. because of heart transplantation. All ICDs implanted for primary prevention in this paediatric cohort were in patients older than 7 years (*Figure 1B*).

### Appropriate ICD therapy

During a median follow-up of 9 years (IQR 4.7–13.5), 44 paediatric patients representing 69% of the total study cohort experienced at least one appropriate therapy. The 1-year cumulative incidence of first appropriate therapy for the total study cohort was 24% (95% CI: 14–33%) (see Supplementary material online, *S2*), with the median time to first appropriate therapy being 5.1 years. The 10-year cumulative incidence for the total study cohort was 69% (95% CI: 56–81%).

**Figure 2:**
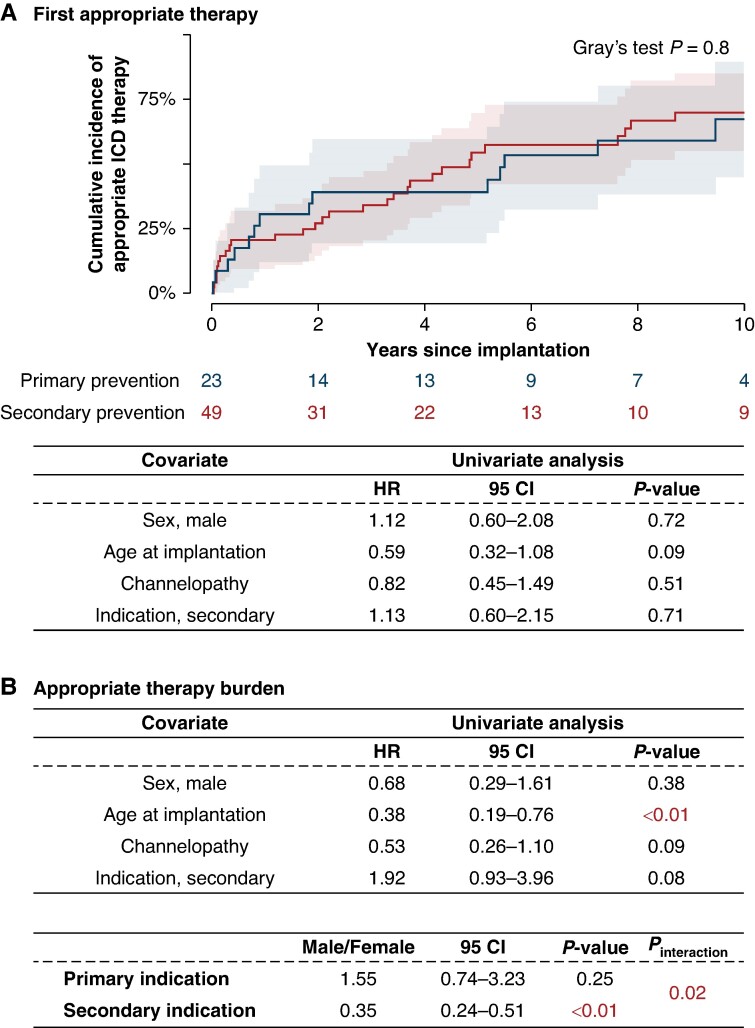
Appropriate ICD therapy. (*A*) Cumulative incidence curve of first appropriate ICD therapy stratified by prevention group. (*B*) Risk factors for the burden of appropriate ICD therapy. The presented HRs for age correspond to age used as a categorical variable with the median as a cut-off. CI, confidence interval; HR, hazard ratio; ICD, implantable cardioverter defibrillator.

There was no significant difference in time to first appropriate therapy for primary vs. secondary prevention groups (*P* = 0.8). Median time to first appropriate therapy was 5.5 years and 4.8 years, respectively, *Figure 2A*. When the four patients categorized as secondary prevention with syncope and a positive EP study with either VT or VF induced were reclassified as primary prevention, there was still no significant difference in time to first appropriate therapy (*P* = 0.6), Supplementary material online, *S3*.

Furthermore, no significant association was found between the time to the first appropriate therapy and whether the implanted ICD was a single-chamber or dual-chamber system (*P* = 0.58). The same applied when stratifying patients with congenital heart disease vs. cardiomyopathies (*P* = 0.41).

In the univariate analysis, neither sex, age, underlying cardiac diagnoses, nor indication group was associated with the risk of first appropriate therapy, *Figure 2A*. However, when assessing age as a continuous variable in the univariate analysis, older age at index implantation was associated with a decreased risk of first appropriate therapy (hazard ratio (HR): 0.91 [0.85–0.98], *P* = 0.02).

A total number of 154 appropriate therapies were delivered during the follow-up. Most therapies were ICD-shocks (*n* = 130, 84%), while anti-tachycardia pacing only accounted for *n* = 24 (16%) of therapies. Among patients with multiple episodes, the maximum episodes per patient reached 29 episodes. Comparatively, 73% of patients receiving appropriate therapy had 1–3 separate episodes. Two-thirds (*n* = 30, 61%) of patients with more than one episode of appropriate therapy were recipients of secondary prevention ICDs. Structural heart disease and ion channel disease were equally presented among patients with multiple appropriate therapies.

Incidence rates of all outcomes obtained from the recurrent events model analysis are presented in Supplementary material online, *S4*.

Age at index ICD implantation was the only covariate associated with recurrent appropriate therapy (*Figure 2B*). When assessed as a categorical variable, higher age was associated with a reduced risk of recurrent appropriate therapies (HR: 0.38 [0.19–0.76], *P* < 0.01), and it remained significant when it was assessed as a continuous variable (HR: 0.88 [0.80–0.97], *P* < 0.01).

Explorative interaction analysis showed that for patients with an ICD implanted for secondary prevention, male gender was associated with a reduced risk of appropriate therapy compared to female gender (HR: 0.35 [0.24–0.51], *P* = 0.02) (*Figure 2B*).

### ICD-related complications

In total, 56 ICD-related complications occurred in 32 out of 72 (41%) paediatric patients during follow-up (see Supplementary material online, *S5*). Lead-related complications were most common during the first 30 days of any implantation. The crude numbers are presented in Supplementary material online, *S6*.

**Figure 3:**
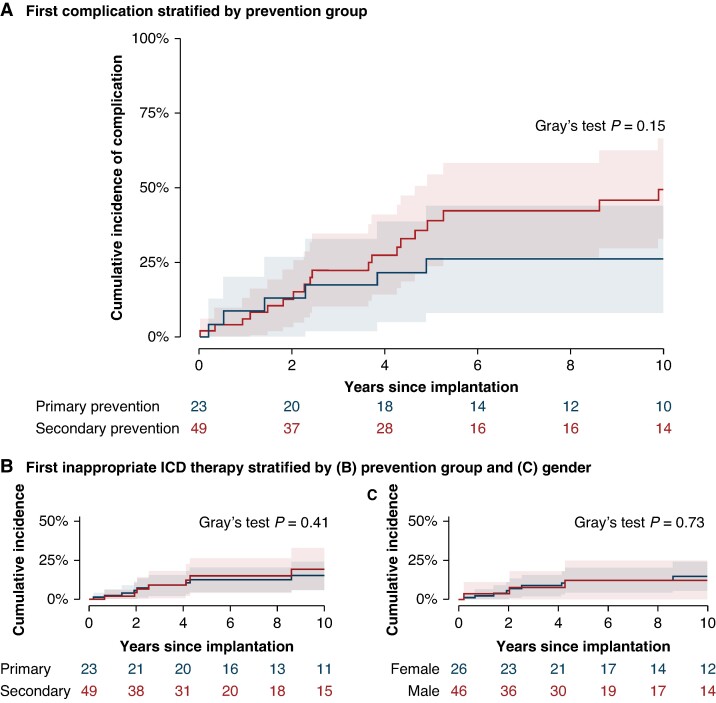
Complications and inappropriate ICD therapy. (*A*) Cumulative incidence curve of complications by prevention group. Complications consist of arrhythmia causing mortality, device-related infections, and other lead-related complications. (*B*) Cumulative incidence curve of first inappropriate ICD therapy stratified by prevention group and (*C*) by sex. ICD, implantable cardioverter defibrillator.

There was no difference in the complication rates between the two prevention groups (*Figure 3A*). Epicardial systems were associated with a significantly lower freedom from complications, *P* < 0.001 (see Supplementary material online, *S7*) predominantly due to a high lead complication rate.

### Inappropriate shock therapy

During follow-up, nine patients (12.5%) received 13 inappropriate shock therapies in total. The causes of inappropriate shocks included sinus tachycardia (*n* = 5), atrial tachycardia (*n* = 3), and unknown (*n* = 5). All incidences led to action in terms of prolonged detection time, increased rate cut-off for detection of ventricular tachycardia, more aggressive anti-arrhythmic medication, electrophysiologic procedure, or atrial tachycardia ablation. Two inappropriate therapies occurred within the first year. The 10-year cumulative incidence of inappropriate shock therapy was 15% for the total cohort (see Supplementary material online, *S8*), with no difference between the two prevention groups (*Figure 3B*) or the sexes (*Figure 3C*). Dual-chamber ICD was not significantly associated with a reduced risk of inappropriate shock therapy compared to single-chamber ICD (HR = 0.86, 95% CI 0.11–6.86, *P* = 0.89).

### Survival

During follow-up, six (8.3%) patients died. Only one patient died in the primary prevention group (*Table 3*, Patient 1). For the arrhythmia-related deaths, three out of four patients had transvenous systems with a range from index implantation to death of 9 days to 17 years. In one patient (*Table 3*, Patient 4), the ICD had correctly detected and treated an episode of VF with one shock therapy, however, relapse of VF was not detected by the ICD due to under-sensing with a fatal result. During testing of the system, there was sufficient detection of VF with successful conversion to sinus rhythm with 20 J ICD-shock. The ICDs acted according to the setting in all cases without ICD system failure. The defibrillation function of the ICD systems was tested after implantation in 63 of 72 patients with success. Another patient died of heart failure despite correctly detected VT (*Table 3*, Patient 6). *Table 3* shows causes of mortality for all six patients. The 10-year survival rate was 94% for the total cohort, with no difference between primary and secondary recipients, *P* = 0.33.

**Table 3:** Mortality, causes of death during follow-up

Patient	Age	Indication	Cardiac diagnosis	Cause of death
1	11	Primary	Congenital long QT	Uncontrollable bleeding in ENTarea. Not related to device.
2	14	Secondary	Congenital long QT	Cardiac arrest with VF. ICD implantation. Anoxic brain injury related to cardiac arrest. ICD is deactivated. Hospice and death.
3	14	Secondary	Truncus arteriosus communis	Rupture of pulmonal artery during pulmonal valve replacement. Anoxic brain injury. Vegetative status leading to death.
4	5	Secondary	Dilated cardiomyopathy	SVT during activity converted to VF. Correctly detected and treated. Shortly after low-amplitude VF that is undersensed.
5	0	Secondary	Cardiac tumour	VT storm. ICD is deactivated after repetitive shocks due to poor prognosis with untreatable tumour.
6	11	Secondary	Ischaemic heart disease	Death because of heart failure despite correctly treated VT.

ENT, ear–nose–throat; ICD, implantable cardioverter defibrillator; SVT, supraventricular tachycardia; VF, ventricular fibrillation; VT, ventricular tachycardia.

## Discussion

Unlike previous ICD studies, this national study consisted solely of paediatric patients ≤ 15 years of age at ICD implantation receiving an ICD between 1982 and 2021. It showed an increasing ICD implantation rate over time in paediatric patients with primary prevention ICDs in children > 7 years old. Two-thirds of the implantations were secondary prevention. A minimum of one appropriate device therapy was experienced by 69% over a median follow-up of 9 years, and the 10-year cumulative incidence of inappropriate therapy was 15% without difference between primary or secondary prevention groups.

The growing ICD implantation rate is not surprising in accordance with already published literature.^1,2^ This can in part be explained by the broader indications, improved survival among paediatric patients with congenital heart diseases,^20^ and advancement in genetic testing. During the last decade, both genetic testing and cardiac magnetic resonance have been available diagnostic tools but with varying implications since the decision of ICD implantation still includes a multifactorial assessment. Additionally, progress in ICD technology for the youngest paediatric patients remains limited, and implanting ICDs in this group requires experience and collaboration with thoracic surgeons. In Denmark, the tradition has been that device implantation was only performed in two centres. However, in recent years, this practice has been further centralized to include only one centre. The surgeons and electrophysiologists treating children have extensive experience with adult patients with congenital heart diseases and maintain a high volume of procedures in adult patients.

The majority of paediatric patients received an ICD for secondary prevention. This differs from the Danish adult ICD recipients with a preponderance of primary prevention ICDs.^7^ A secondary prevention ICD is indicated according to guidelines after an aborted cardiac arrest or documented malignant arrhythmia when reversible causes have been excluded. In these cases, ICD implantation is indicated regardless of the patient’s age. However, without an event, the threshold for ICD implantation for primary prevention in paediatric patients is probably higher due to a lack of guidelines. Previous paediatric studies on ICDs did not find the same distribution of primary and secondary ICDs as the present study.^1,21^ This could be because they only included patients in partial periods of our entire study period, and their study populations were defined as <21 and <18 years, respectively. Different geographies and healthcare systems can be expected to have different practices, which can contribute to the differences.

The high incidence of appropriate therapy in this cohort can be explained by the selection process since all the included patients are high-risk patients. Of the ICD recipients with CPVT or congenital long QT syndrome, the prevalence of anti-arrhythmic medication is 76% supporting that these patients are high risk. The patients not on beta-blockers were all initiated and eventually, ceased treatment due to intolerable side effects. Even though anti-arrhythmic medication is commonly used, it does not necessarily equate to adequate medication or compliance. The high proportion of shock therapies compared to ATP treatment in our study supports that ICDs are programmed to treat VF or very fast VT only (as a ‘shock box’), since ventricular tachycardia susceptible to ATP therapy is rare in paediatric patients, whereas the risk of inappropriate therapy is increased.

The equal rate of appropriate therapy for primary vs. secondary recipients is similar to another study.^22^ This study included 99 children with a median follow-up of 4.9 years and found no statistical difference in the incidence of appropriate shock rates among primary and secondary recipients. The incidences were 32% and 48%, respectively. In contrast, studies including young adults aged <30 years reported a higher incidence of appropriate therapy in secondary recipients compared to primary recipients.^23,24^ The inclusion of adults will decrease the overall therapy burden. For secondary prevention, our incidence of appropriate ICD therapies is comparable with that observed in adults with secondary prevention ICDs.^25^

There was a sex difference in paediatric patients receiving appropriate therapy for secondary prevention. Sex hormones are believed to play a role in arrhythmic events in long QT syndrome patients, who account for the majority of our primary recipients.^26^ A prior study^27^ suggests that male paediatric patients > 16 years old have a lower probability of cardiac events, which may also be a factor in our study. However, we lacked genetic data, limiting statistical power to demonstrate this.

We found a 10-year inappropriate therapy rate of 15%, comparable to adult populations,^28^ which is consistent with previous research in the paediatric populations.^6,21–24,29–36^ One study that stratified therapies by indications reported no difference after 5 years^23^ consistent with our results with longer follow-up. Our results indicate that improved monitoring, device programming, medical treatment, and AT-ablation may have led to the reduced incidence of inappropriate therapies. Additionally, since children have physiologically higher heart rates compared to adults, we do program the ICDs individually and conservatively based on age and with a high heart rate threshold to avoid inappropriate therapies. This is further supported by our experience that VF episodes occur more often than VT episodes.

The high incidence of appropriate and low incidence of inappropriate therapy indicates that current practice results in careful selection of patients with a high sensitivity. Often, a high sensitivity is at the cost of the specificity and certainly raises concerns that there are arrhythmic deaths occurring in children that could have been prevented. This issue cannot be assessed with the data available but should be the subject of future studies. The incidence of SCD in this age group is assessed in a study by Risgaard *et al*.^37^ to be <1/200 000 persons/year, which complicates research in this area. Only 35% have symptoms before SCD and 22% have been found to have a contact with the healthcare system in close proximity to experiencing SCD,^38^ which further complicates the topic. However, these are retrospective studies and data are often missing. This highlights the need for careful prospective studies. Implantable cardiac monitors^39^ and wearables are promising tools for future studies, but there is limited published experience in the field.^40^ Until then, our data give re-assurance that the current practice provides an ICD to children at high risk for malignant arrhythmias carrying a high yield of ICD implantation where a substantial complication burden is justified.

### Limitations and strengths

For a rare condition requiring a high degree of specialization, a large sample size is required to make a robust conclusion. This was a small retrospective uncontrolled study with a heterogeneous patient group. By including national data with long follow-up, we tried to overcome the sample size issue. Additionally, this national cohort reports on consecutive data for a 30-year period. Since this treatment in children is extremely rare, large cohorts will be rare.

Also, given the observational nature of the present study, no causal inference can be made from our results, although all baseline characteristics and study outcomes have been validated through medical charts. Furthermore, due to heterogeneity, caution should be paid when comparing studies. In this study, we did not include the patients discussed by the multidisciplinary team who did not receive an ICD, which could have strengthened the results.

Several studies have investigated cohorts of both paediatric and adult patients with structural heart disease.^5,41–43^ However, this is one of the few studies reporting outcomes in an exclusively paediatric cohort, who are expected to have longer life expectancy and multiple device changes during their lifetime. Thus, prospective multicentre studies may be desired. Although the data presented here are retrospective, this is a multicentre study using a validated national database linked to hospital records providing greater data granularity than many registry-based studies.

## Conclusion

ICD implantation in paediatric patients is rare but has increased over the last decade. One-third of paediatric implantations are for primary prevention. The incidence of appropriate ICD therapy is high with a 10-year cumulative incidence of 69% and comparable for primary and secondary prevention recipients. The rate of appropriate therapy was higher than reported for adult populations. Additionally, the complication rate was high without difference for the two prevention groups, while the incidence of inappropriate shock therapies was low. These findings support that the identified ICD recipients were benefitting from the ICD implantation. To what extent the restrictive primary prevention indication practice results in missing preventable arrhythmic deaths remains uncertain. Nevertheless, our data emphasize the necessity for future trials focusing on risk stratification and primary prevention of arrhythmic death.

## Supplementary Material

euae245_Supplementary_Data

## Data Availability

The data underlying this article cannot be shared publicly, as in compliance with the General Data Protection Regulation.
