# Supplementary material for: Implantable cardioverter defibrillator therapy in paediatric patients for primary vs. secondary prevention
Source: Europace. 2024 Sep 30;26(9):euae245. doi: 10.1093/europace/euae245 (PMC11440178; doi:10.1093/europace/euae245)

# **Supplementary data**

**S1;** Prevalence of beta-blocker treatment among the 34 patients with CPVT or congenital long QT syndrome

|  | All | Primary prevention | Secondary prevention |
| --- | --- | --- | --- |
|  |  |  |  |
| Beta-blocker | 26/34 (76 %) | 12/13 (92 %) | 14/21 (67 %) |

**S2;** Cumulative incidence of appropriate therapy in the whole cohort


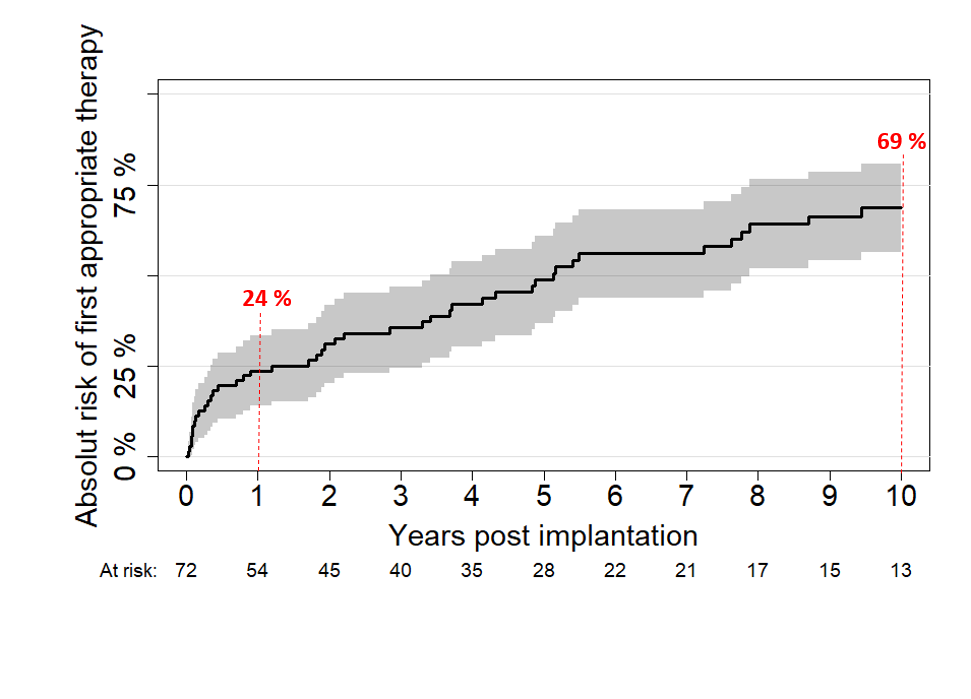


**S3;** Sensitivity analysis for time to first appropriate ICD therapy in the re-defined primary and secondary prevention group


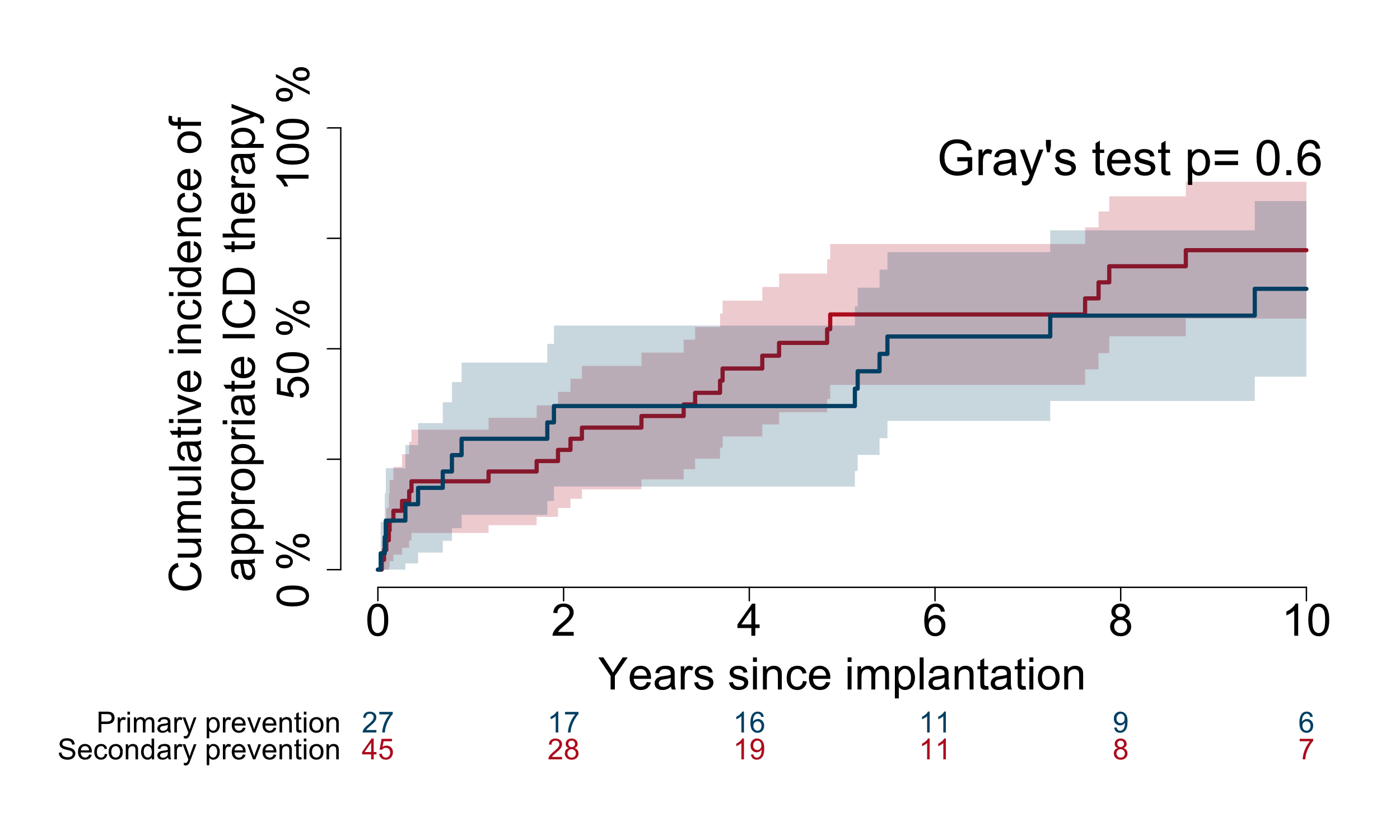


**S4;** Incidence rates of events with recurrent events

|  | **Percentual incidence**  **in the cohort (%)** | **Number of events** | **Incidence rate/100 patient years*** |
| --- | --- | --- | --- |
| Appropriate therapy | 69 | 154 | 22 |
| Complications | 41 | 56 | 8 |
| Inappropriate therapy | 15 | 13 | 2 |

**The total follow-up was 692 patient-years.*

Complications consist of arrhythmia causing mortality, device-related infections, and other lead-related complications

**S5;** Cumulative incidence of complications in the whole cohort


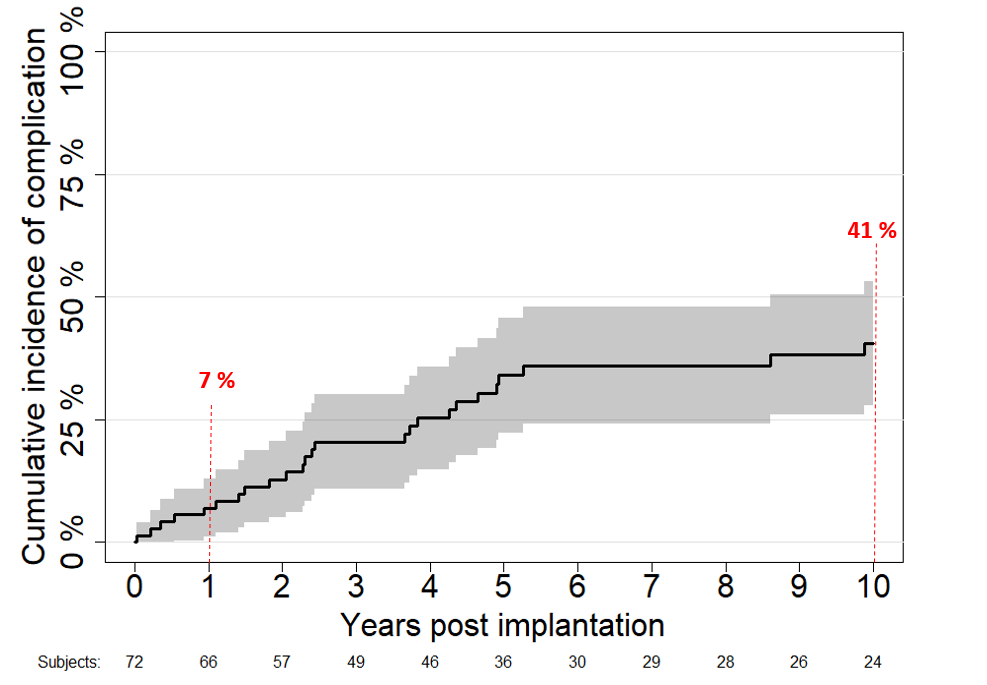


**S6;** Number of different outcomes divided into procedural (≤30 days of *any* implantation) or non-procedural (>30 days after *any* implantation) stratified by prevention group. One patient can be represented multiple times in case of multiple re-operations

**
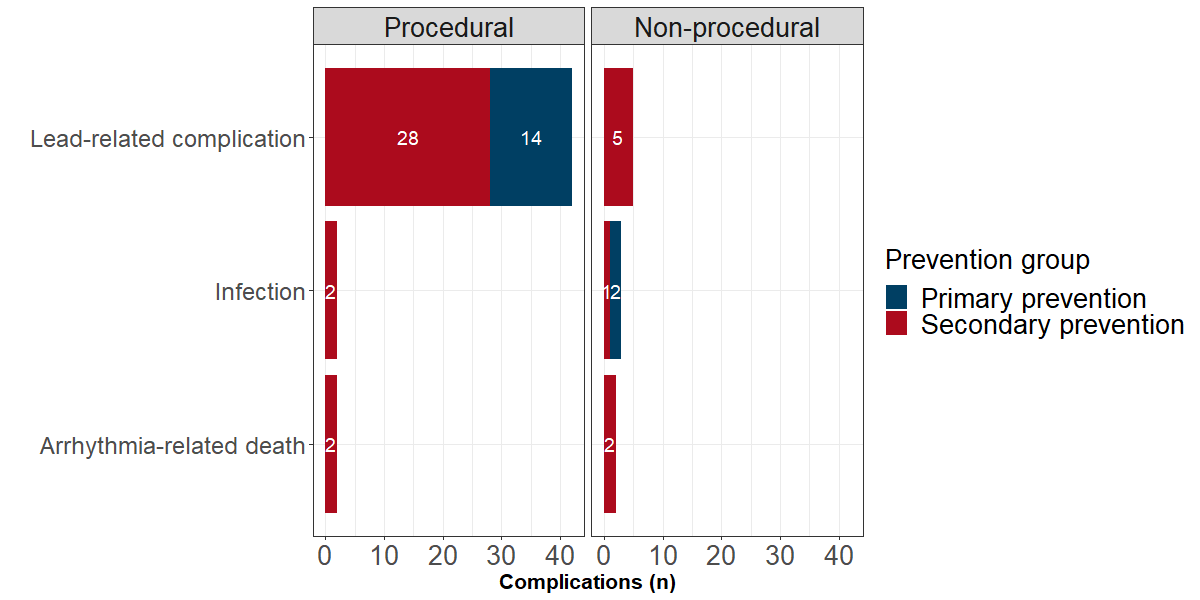
**

**
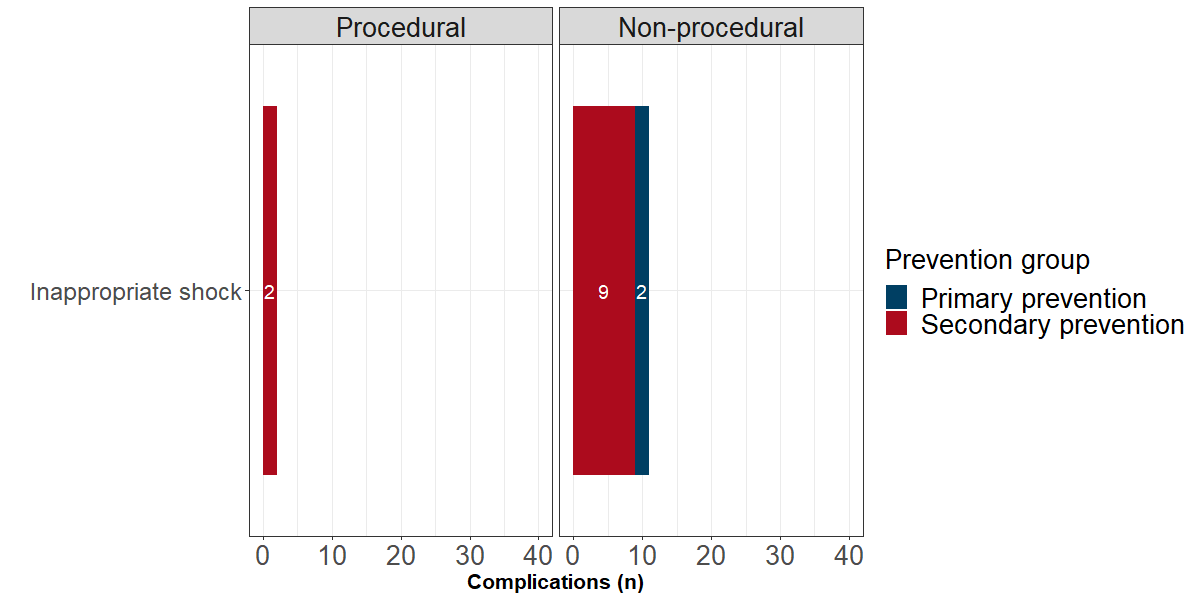
**

**S7:** Cumulative incidence of complications stratified by implantation technique

**S8;** Time to inappropriate shock therapy in the whole cohort


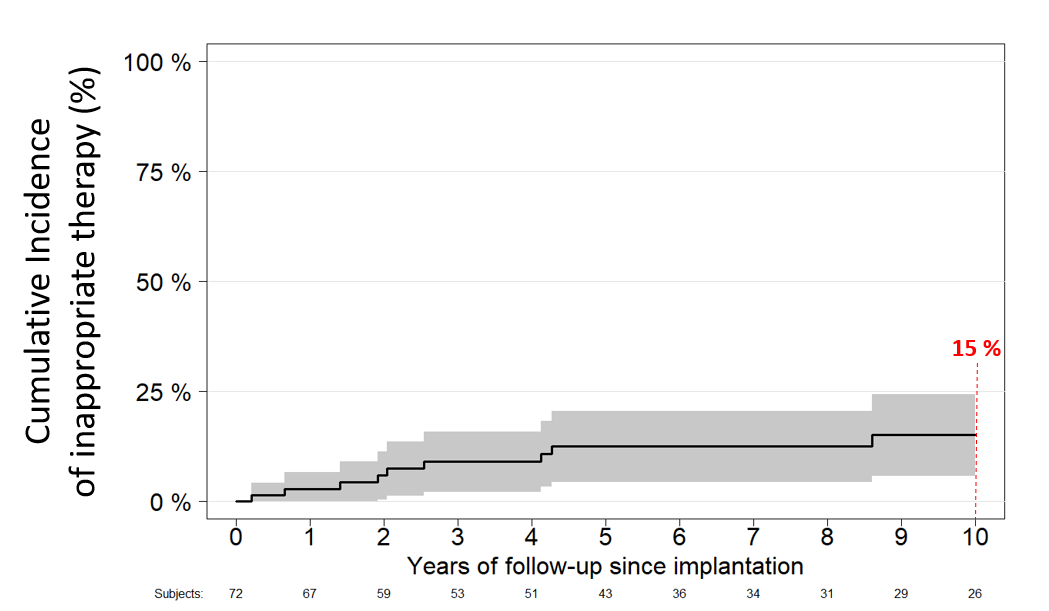

Supplement: euae245_Supplementary_Data [file euae245_supplementary_data.docx]
